# Draft genome sequences of two *Pseudomonas* species from Lake St. Clair (Ontario, Canada) encode multiple heavy metal resistance determinants

**DOI:** 10.1128/mra.00483-26

**Published:** 2026-06-15

**Authors:** Xuexing Yao, Kate Brown, R. Michael McKay, Opeyemi U. Lawal

**Affiliations:** 1 Great Lakes Institute for Environmental Research, University of Windsor 8637, Windsor, Ontario, Canada; 2 School of the Environment, University of Windsor 8637, Windsor, Ontario, Canada; 3 Centre for Great Lakes and Watershed Studies, Bowling Green State University, Bowling Green, Ohio, USA; 4 WE SPARK Health Institute 712165, Windsor, Ontario, Canada; Portland State University, Portland, Oregon, USA

**Keywords:** freshwater ecosystems, antimicrobial resistance, heavy metal resistance, Great Lakes, One Health

## Abstract

Freshwater ecosystems represent dynamic habitats influenced by non-point source agricultural discharge and industrial inputs, providing an interface for examining antimicrobial resistance determinants within a One Health framework. Here, we report the draft genome sequences of two *Pseudomonas* species recovered from Lake St. Clair encoding multiple heavy metal resistance determinants.

## ANNOUNCEMENT

Freshwater ecosystems in the Great Lakes region are influenced by urban, agricultural, and industrial discharges, which can render these systems hotspots for antimicrobial resistance (AMR) determinants (1, 2). Lake St. Clair is a transnational freshwater system that represents a One Health interface, where environmental contamination may affect ecosystem health as well as human and animal exposure (3). *Pseudomonas* species are widespread in aquatic environments and resilient under contaminant stress with a tendency to harbor multiple AMR determinants (4). As part of assessing the AMR dynamics in freshwater systems, two *Pseudomonas* species were isolated and characterized.

Freshwater samples (1 L) were collected in Lake St. Clair, Ontario, using an integrated sampler extending to 2 m depth (5), and transported on ice until processing. Sample details are provided in Table 1. Briefly, aliquots (100 µL) were plated onto MacConkey Agar (Becton, Dickinson and Co., MD, USA) and incubated for 24–48 h at 37°C to promote the growth of mesophilic and potential enteric bacteria. Distinct single bacterial colonies of differing morphologies were sub-cultured multiple times onto tryptic soy agar and incubated for 24–48 h at 37°C. DNA was extracted from pure colonies on plates using the DNeasy Blood and Tissue Kit (Qiagen Germantown, MD, USA) following manufacturer’s instructions. DNA libraries were prepared using tagmentation with Integrated DNA Technologies (IDT) for DNA/RNA UD Indexes (Illumina, San Diego, CA, USA) (6) and sequenced using 2 × 300 bp paired-end chemistry on an Illumina NextSeq 1000 instrument at the University of Windsor Environmental Genomics Facility.

**TABLE 1:** Sequencing metrics for two *Pseudomonas* species isolated from Lake St. Clair, Ontario

ID	*P. simiae* Env_E2	*P. siliginus* Env_H1
Collection date	30 July 2025	30 July 2025
Source	Belle River	Stony Point
GPS coordinates	42.30111,−82.70555	42.32166,−82.55195
ECCC^*a*^ station number	EC 135	EC 136
Length	6,318,914	6,129,665
Coverage	93.07	85.08
# Contigs	80	52
Genome size	6,318,914	6,129,665
% GC	60.24	59.73
N50	136,268	269,705
% Genome completeness	100	100
% Contamination	0.3	0.2
Accession #	SRR37250440	SRR37250441
BioSample #	SAMN55383886	SAMN55383879
Assembly #	JBVHCW000000000	JBVHCX000000000
Genes	5,896	5,617
CDS	5,826	5,549
Genes encoding protein	5,692	5,463
Pseudogene	134	86
Transfer RNA	60	59

^*a*^ ECCC, Environment and Climate Change Canada.

Isolates Env_E2 and Env_H1 yielded 7,187,016 and 21,064,893 reads, respectively. Reads were quality-checked using FastQC v0.11.9 (https://github.com/s-andrews/FastQC) and then trimmed using Trimmomatic v0.39 (7). Reads with Phred score of >20 were assembled *de novo* using SKESA v2.4.0 (8). Assembly quality and genome completeness were assessed using QUAST v5.2 (9) and CheckM2 v1.1 (10). Genome annotation was performed using the National Center for Biotechnology Information Prokaryotic Genome Annotation Pipeline v6.10 (11). Draft genomes were screened for AMR using AMRFinder Plus v4.2.6 (12). Default parameters were used for all bioinformatics tools.

Taxonomic classification of Env_E2 and Env_H1 using Type (Strain) Genomic Server (TYGS) (13, 14) identified them as *Pseudomonas simiae* and *Pseudomonas siliginis*, respectively. *P. simiae* Env_E2 had a genome size of 6,318,914 bp, 80 contigs with N_50_ of 136,268 bp, 93× mean coverage, and 60.24% GC content. Comparatively, *P. siliginis* Env_H1 had a genome size of 6,129,665 bp, 52 contigs with N_50_ of 269,705 bp, 85× mean coverage, and 59.73% GC content. Other sequence metrics and annotation data are listed in Table 1. Both *Pseudomonas* strains carried genes predicted to encode antimicrobial resistance protein (*mig-14*) and multiple heavy metal resistance determinants, including those encoding resistance to copper (*copI* and *mco*), arsenic (*arsH*), and tellurite (*terB*). *P. siliginis* Env_H1 carried additional resistance determinants, including mercury resistance operon (*merA*, *merC*, *merD*, *merE*, and *merT*) and fosfomycin resistance gene (*fos*), while bleomycin resistance protein was detected only in *P. simiae* Env_E2.

Overall, these genomic data sets will contribute to the evolutionary understanding of these *Pseudomonas* species and to AMR monitoring in freshwater ecosystems at the One Health interface.

## Data Availability

This whole genome shotgun project has been deposited at DDBJ/ENA/GenBank under the BioProject accession PRJNA1424412. The assemblies and annotation accessions are listed in Table 1.

## AFTER EPUB

*[This article was published on 15 June 2026 with an error in the affiliations. The affiliations were corrected in the current version, posted on 22 June 2026.]*
